# Parameter balancing: consistent parameter sets for kinetic metabolic models

**DOI:** 10.1093/bioinformatics/btz129

**Published:** 2019-02-21

**Authors:** Timo Lubitz, Wolfram Liebermeister

**Affiliations:** 1 Theoretische Biophysik, Institut für Biologie, Humboldt-Universität zu Berlin, Berlin, Germany; 2 INRA, UR1404, MaIAGE, Université Paris-Saclay, Jouy-en-Josas, France; 3 Institut für Biochemie, Charité, Universitätsmedizin Berlin, Berlin, Germany

## Abstract

**Summary:**

Measured kinetic constants are key input data for metabolic models, but they are often uncertain, inconsistent and incomplete. Parameter balancing translates such data into complete and consistent parameter sets while accounting for predefined ranges and physical constraints. Based on Bayesian regression, it determines a most plausible parameter set as well as uncertainty ranges for all model parameters. Our tools for parameter balancing support standard model and data formats and enable an easy customization of prior distributions and constraints for biochemical constants. Modellers can balance kinetic constants, thermodynamic data and metabolomic data to obtain thermodynamically consistent metabolic states that comply with user-defined flux directions.

**Availability and implementation:**

An online tool for parameter balancing, a stand-alone Python command line tool, a Python package and a Matlab toolbox (which uses the CPLEX solver) are freely available at www.parameterbalancing.net.

## 1 Introduction

Kinetic models are important tools for understanding metabolic dynamics. A main challenge in model construction is the choice of rate laws and kinetic parameters, such as Michaelis–Menten constants, catalytic rate constants or equilibrium constants. Network models can be populated automatically with kinetic rate laws (Dräger *et al.*, 2008; Liebermeister and Klipp, 2006a) and parameter values obtained from repositories, such as BRENDA, SABIO-RK or eQuilibrator (Flamholz *et al.*, 2012). However, values may be unreliable due to experimental errors, *in-vitro* measurements, measurements in other organisms or differing experimental setups. When inserted into a model, such measured or calculated parameters may cause inconsistencies. For example, the thermodynamic Wegscheider conditions and Haldane relationships create physical dependencies between different kinetic constants. Ignoring these dependencies may lead to thermodynamically incorrect models that describe, effectively, a *perpetuum mobile*. In theory, missing parameters can be determined by fitting a model to metabolomic time-series data. However, parameter fitting can be numerically hard, parameters may not be identifiable, and in practice the thermodynamic dependencies between parameters are often ignored.

Parameter balancing (Lubitz *et al.*, 2010) is a Bayesian parameter estimation method that addresses these problems. It converts kinetic and thermodynamic constants, which may be uncertain and incomplete, into a consistent set of model parameters. Metabolic network structure and known thermodynamic constraints define dependencies between all model parameters. To respect these dependencies, the parameters are considered on logarithmic scale and expressed as linear functions of a smaller number of independent basic parameters. With data on some parameters, prior distributions, and presumed parameter ranges, the estimation of these basic parameters becomes a linear regression problem to be solved in a Bayesian framework. The result is a multivariate posterior distribution describing the mean values, uncertainties and correlations of all model parameters. Aside from obtaining point estimates and uncertainty ranges for single parameters, one may sample parameter sets from the posterior to construct an ensemble of model variants that agree with all available knowledge and data, and the posterior may be used as a prior in subsequent rounds of model fitting (Liebermeister and Klipp, 2006b). Aside from kinetic constants, also metabolite concentrations, flux directions and thermodynamic forces can be obtained for estimations of metabolic states.

## 2 Results and implementation

To make parameter balancing applicable, we provide software tools as well as Python and Matlab code. Our online interface allows modellers to balance model parameters with a few mouse clicks while the Python and Matlab packages can be integrated into modelling workflows (Smith *et al.*, 2018; Stanford *et al.*, 2013). Our implementation supports established standard formats: models are given in Systems Biology Markup Language format while the generic table format SBtab (Lubitz *et al.*, 2016) is used for all other data. Based on a network model and kinetic parameter data, the tool returns a table with balanced parameter values and their uncertainties as well as a parameterized Systems Biology Markup Language model with modular rate laws (Liebermeister *et al.*, 2010). Parameter balancing can be customized by modifying the priors, e.g. by specifying new mean values, standard deviations and possible ranges for various types of kinetic constants. Metabolic fluxes cannot be used directly as input data because typical kinetic rate laws do not fit into the regression model underlying parameter balancing. However, if flux directions are known, they can be used to define the signs of thermodynamic forces, and the resulting metabolic state will comply with these flux directions. In the Matlab version, enzyme levels and catalytic constants can be automatically adjusted to match predefined fluxes; moreover, parameter sets can be sampled from the posterior distribution, where corrections are applied if parameters violate predefined bounds. In a model with 50 reactions, the calculation takes less than a second on a 2.7 GHz CPU. To restrict the numerical effort, model sizes are limited to 250 reactions, and for large models, using the Matlab version of our tool with CPLEX solver is recommended.

## 3 Conclusion

**Fig. 1.**
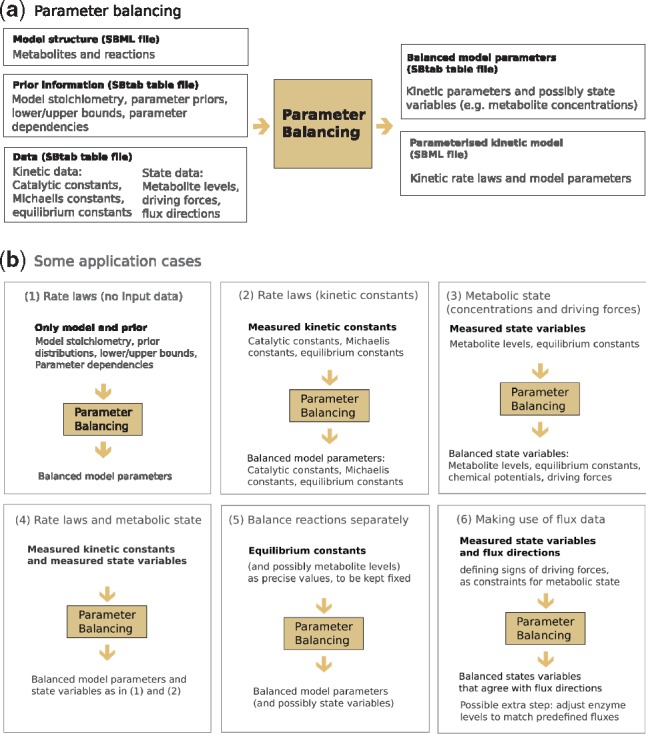
Parameter balancing. (**a**) Input and output data. (**b**) Use cases: (1) Finding realistic kinetic parameters for a given network model without any measurement data. Parameters are determined from prior distributions, known dependencies, and upper and lower bounds. (2) Translating incomplete kinetic data into balanced model parameters. (3) Predicting realistic metabolic states (including metabolite levels, equilibrium constants, chemical potentials and thermodynamic forces) from measured metabolite levels and equilibrium constants. (4) Combining points (2) and (3) to determine metabolic state, balanced kinetic constants and rate laws simultaneously. (5) To speed up calculations, parameters can be balanced separately in each reaction. Equilibrium constants and metabolite levels need to be set in advance to obtain a consistent model. (6) If metabolic fluxes are known, the flux directions can be used to constrain the possible metabolic states, and enzyme levels can be adjusted to match the predefined fluxes

Parameter balancing translates metabolic network structures into dynamic models, using experimental data collected from the literature. As shown in Figure 1, it allows modellers to find plausible default parameters for a model (even without any data, and based on the priors), to balance given kinetic constants, or to determine consistent metabolite concentrations and thermodynamic forces based on predefined flux directions. In all cases, adding more data improves the accuracy of the balanced parameters and reduces their uncertainty ranges.
